# Nutrient Storage in the Perennial Organs of Deciduous Trees and Remobilization in Spring – A Study in Almond (*Prunus dulcis*) (Mill.) D. A. Webb

**DOI:** 10.3389/fpls.2020.00658

**Published:** 2020-06-23

**Authors:** Saiful Muhammad, Blake L. Sanden, Bruce D. Lampinen, David R. Smart, Sebastian Saa, Kenneth A. Shackel, Patrick H. Brown

**Affiliations:** ^1^Department of Plant Sciences, University of California, Davis, Davis, CA, United States; ^2^UC ANR Cooperative Extension, University of California, Bakersfield, Bakersfield, CA, United States; ^3^Department of Viticulture and Enology, University of California, Davis, Davis, CA, United States; ^4^Almond Board of California, Modesto, CA, United States

**Keywords:** nitrogen, potassium, nutrient, perennial organs, storage, remobilization

## Abstract

The annual dynamics of whole mature almond tree nutrient remobilization in spring and the accumulation of nutrients in perennial tissues during the year were determined by sequential coring, tissue sampling, nutrient analysis, whole tree excavation and biomass estimation for trees grown under four nitrogen rate treatments 140 kg ha^−1^ N (N140), 224 kg ha^−1^ N (N224), 309 kg ha^−1^ N (N309), and 392 kg ha^−1^ N (N392) over 2 years. Whole tree perennial organ N content was greatest in dormancy then declined through bud swell, flowering and fruit set, achieving the lowest total whole tree nutrient content of perennial organs by March 12 [12–14 days after full bloom (DAFB)] coincident with 60–70% leaf expansion. During this period no net increment in whole tree N content (annual plus perennial N) was observed indicating that tree demand for N for bud break, flowering, fruit set and leaf out was met by remobilized stored N and that there was no net N uptake from soil. Remobilizable N increased with increasing N application up to N309 and was maximal at 44.4 ± 4 kg ha^−1^ and 37.5 ± 5.7 kg ha^−1^ for the optimally fertilized N309 in 2012 and 2013 respectively. Net increases in perennial organ N (stored N) commenced 41 DAFB and continued through full leaf abscission at 249 DAFB. Total annual N increment in perennial organs varied from 25 to 60 kg ha^−1^ and was strongly influenced by N rate and tree yield. N remobilized from senescing leaves contributed from 11 to 15.5 ± 0.6 kg ha^−1^ to perennial stored N. Similar patterns of nutrient remobilization and storage were observed for P, K, and S with maximal whole tree perennial storage occurring during dormancy and remobilization of that stored P, K, S to support annual tree demands through to fruit set and 70–100% leaf development. Net annual increment in perennial organ P, K, S commenced 98 DAFB and continued through full leaf abscission at 249 DAFB. Organ specific contribution to remobilizable and stored nutrients changes over the growing season are presented. Details of the pattern of perennial organ nutrient allocation, storage, and remobilization provides a framework for the optimal management of nutrients in almond with relevance for other deciduous tree species.

## Introduction

Deciduous trees cycle nitrogen (N) and other nutrients by remobilizing them from the senescing leaves into woody tissue and by storing a portion of accumulated nutrients in perennial organs. Subsequently, stored nutrients are preferentially utilized for growth in the spring (Kang et al., 1982; Titus and Kang, 1982; Tromp, 1983; Millard and Thomson, 1989; Tagliavini et al., 1998; Millard and Grelet, 2010). Millard (1988) defined N storage as nitrogen that could be remobilized from one tissue for the growth or maintenance of another. The size of the storage pool is therefore defined by the net change in nutrient content in an organ over time. Deciduous species tend to store N in the wood and bark of roots or the trunk (Millard and Grelet, 2010). Unlike many deciduous tree species, flowering in almond normally begins before leaf emergence, thus flowering and early fruit growth likely depends primarily on stored nutrients. Stored nutrients play a pivotal role in the nutrition of the tree at a time when soil nutrient uptake is limited by environmental factors, lack of transpiration and restricted root growth (Basile et al., 2007). N is stored in trees in a wide range of different vegetative storage proteins, including as bark storage proteins (Cooke and Weih, 2005). Initial tree growth in the spring utilizes remobilized N rather than N uptake by roots due to suboptimal conditions, abundance of stored N and absence of leaves (Millard and Neilsen, 1989; Millard and Grelet, 2010).

Nutrient storage has been inferred in several manners (a) determination of the difference in nutrient concentration in perennial tissues between dormancy and lowest content observed following spring growth using ^15^N (Deng et al., 1989), (b) analyzing ^15^N in annual tissues from previous year’s ^15^N application (Weinbaum et al., 1984; Weinbaum and Klein, 1986), and (c) nutrient budget studies with young (Miller, 1995; Tagliavini et al., 1999; Kim et al., 2009; Millard and Grelet, 2010) and mature trees (Rosecrance et al., 1998; Acuna-Maldonado et al., 2003). Each of these approaches has limitations. The use of the change (decrease) in nutrient concentrations in dormant versus growing perennial organs, without determining overall biomass, does not provide information on total storage pool (Rosecrance et al., 1998) and in the absence of biomass information the extent to which depletion is occurring cannot be determined. The use of ^15^N in sand culture studies with young trees in pot experiments (Millard and Proe, 1993; Grassi et al., 2002; Guak et al., 2003) or with mature field grown trees in sandy soils (Weinbaum et al., 1984; Weinbaum and Klein, 1986) does not take into consideration ^15^N leaching nor long term N immobilization or exchange rates with native soil N. Nutrient budget studies using young trees have also been questioned as these do not represent mature trees, thus, extrapolation to mature tree introduces considerable uncertainty (Rosecrance et al., 1998).

Nutrient budgets developed using mature tree excavation and determining the perennial biomass and nutrient concentration of tree organs provides a better estimate of the nutrient content and overall storage pool, which can then be calculated as the difference in nutrient content of the perennial organs at a seasonal maximum and minimum (Rosecrance et al., 1998). Here we used both tree excavation at the beginning and end of the season and in-season tree monitoring to track changes in nutrient concentration by taking multiple stem core samples (trunk and scaffold), digging roots and taking samples from branches. We estimated nutrient storage as the difference in tree nutrient content when the tree was dormant, versus that when seasonal minimum was observed. Most of the previous studies have used one standard fertilizer rate and, to our knowledge, the effect of differential N fertilization on storage of N and other nutrients has not been studied. Most studies have also focused on the storage of N and only a few have studied P and K storage while there is no information about the storage of other nutrient elements.

Quantifying the amount of nutrients stored and its remobilization is important in developing fertilization programs and interpreting ecosystem level nutrient budgets. Under environmental conditions where heavy rainfall during early spring may contribute to leaching of N from the soil profile, knowing the dynamics of storage and remobilization can help in adjusting fertilizer application to nutrient demand with soil nutrient uptake.

The objectives of this study were to (1) determine the size of storage pool of N, P, K, S, B, and other elements under different N application levels, (2) To determine which organs are used for nutrient storage in almond trees, (3) to document the timing of nutrient remobilization and storage, and (4) to determine the contribution of leaf nutrient remobilization into the storage pool during senescence. We hypothesize that well-fertilized almond tree stores an appreciable amount of nutrients and that these stored nutrients to support a large proportion of nutrient demand during flower and fruit development and initial leaf growth.

## Materials and Methods

### Experimental Site

The experiment was carried out in a commercial almond orchard near Belridge, Kern County, California (35.5° N 119.6° E). The orchard was planted to 50% Nonpareil and 50% Monterey grafted onto peach rootstock [*Prunus persica* (L.) Batsch cv. Nemaguard] with a planting density of 214 trees per ha. The soil type was Panoche and Kimberlina very fine sandy loam (Loamy, mixed, superactive, calcareous, thermic Typic Torriorthents) with EC of 1.3 ds m^−1^ and pH 7.8 in the top 46 cm and EC 2.2 ds m^−1^ and pH 7.8 in 47–91 cm depth and organic matter of less than 1% with good drainage and aeration (Muhammad et al., 2018). A fertilizer rate trial was established in 2008 when the orchard was 9 years old and maintained through completion of this experiment (Muhammad et al., 2015). N was applied at 140 kg ha^−1^ (N140), 224 kg ha^−1^ (N224), 309 kg ha^−1^ (N309), and 392 kg ha^−1^ (N392) with Urea Ammonium Nitrate 32 (UAN 32). The experiment was laid out in randomized complete block design and each treatment was replicated five times. N was applied in four fertilization events as 20, 30, 30, and 20% of total in mid-February, early April, mid-June and post-harvest, corresponding to flowering and early leaf out, early nut-fill, nut maturation, and the post-harvest period respectively (Muhammad et al., 2015). All the nitrogen treatments received 90 kg ha^−1^ phosphorus as phosphoric acid and 224 kg ha^−1^ potassium as 60% potassium sulfate (SOP) and 40% potassium thiosulfate (KTS). SOP was applied in December in 60 cm wide stripe on both side of the tree rows and was incorporated into the soil by winter rain and irrigation. KTS was applied with irrigation water in four fertilization event as for N above.

Orchards were irrigated using micro sprinkler at 100% ET_*c*_ as determined from two custom surface renewal instrument towers installed onsite (see Shapland et al., 2012). Soil moisture was monitored via neutron probe (Model 503DR, ICT International, Armidale, NSW, Australia) in bore holes established to 2 m. Periodic measures of stem water potential were obtained using a pressure chamber (Model 3005HGPL Soil Moisture, Inc., Santa Barbara, CA, United States) to ensure recommended growth conditions were maintained. Determination of nutrient storage in perennial organs commenced in 2012 4 years after treatment commencement.

### Tree Excavation for Biomass and Nutrient Analysis

Two entire mature trees from each N rate were selected on the basis of their representative yield over the preceding 4 years and excavated in January 2012 to determine the biomass of the different organs and nutrient concentration. Two trees from each N rate treatment were also excavated in January 2013. N392 was discontinued in 2013 and in January 2014, three trees were excavated each from the N140, N224, and N309 treatments. Trees were excavated using a backhoe and separated by hand into trunk, roots < 1 cm diameter (small roots), roots > 1 cm diameter (large roots), scaffold, canopy branches and branches > 2.5 cm diameter. The uniform fine-sandy loam soil in this orchard, a highly restricted root distribution which remained almost entirely within the wetted irrigation zone, and the labor of a large crew of workers, allowed for a very high level of recovery of all but the finest of roots. Biomass of each part was determined as follow:

1. Trunk

The portion of the stem above the crown after removing the scaffold branches was considered the trunk.

2. Roots

The roots were dug out from a 5 m wide x 6 m long x 1.2 m deep monolith area and were separated from the soil using a large crew of laborers. The roots were washed using pressurized water to remove soil. Roots were divided into two groups: roots < 1 cm diameter and root > 1 cm diameter. As excavations were performed during dormancy there were no apparent fine roots (≤ 2 mm diameter) or white root growth and hence it was considered the vast majority of the standing root system was recovered.

3. Scaffold

Scaffold branches that support the functional canopy, were separated from canopy branches.

4. Branches

Canopy branches > 2.5 cm diameter and small branches < 2.5 cm diameter were separated.

Fresh weight of all the organ groups mentioned above were determined in the field. A 5 kg sub sample was collected from all the groups and dried at 70°C air forced driers for 7 days to determine dry weight. Organ dry weight was used to determine total tree dry biomass.

### Sample Collection and Nutrient Analysis

Samples for chemical analysis from each category above were collected from the excavated trees as follow:

1. Trunk

Wood samples for nutrient analysis were collected from the sap wood by boring at multiple points in the trunk using portable drill equipped with wood boring bit and the shavings were collected in bags.

2. Roots

Roots were washed to remove soil. Samples from large roots were collected by boring using drill at multiple location in the roots and shaving were collected in bags. Smaller roots were cut and brought to the lab where they were washed to remove soil.

3. Scaffold

Several bore holes were drilled in the scaffold and wood samples collected in bags for nutrient analysis.

4. Branches

Wood samples were collected by boring into branches at multiple locations and shavings were then collected for nutrient analysis. Samples from small branches < 2.5 cm diameter were collected by cutting branches in small pieces.

All the samples were dried at 60°C for 5 days. The wood shavings collected from trunk, scaffold, and canopy branches were ground using a Wiley Mill (Thomas Scientific, Inc.) to pass a 0.3 mm screen. Samples from roots > 1 cm diameter and branches < 2.5 cm diameter were cut with a manual saw and sawdust was collected. Sawdust was then ground using Wiley Mill to pass 0.3 mm screen. Fine branches and roots < 1 cm diameter were ground using a Wiley Mill. Samples were analyzed for N, P, K, S, B, Ca, Mg, Mn, Zn, Fe, and Cu in the University of California, Davis Analytical Laboratory (ANL). N was determined through combustion (AOAC, 2006) and all other elements through nitric acid digestion and determination by Inductively Coupled Plasma Atomic Emission Spectrometry (ICP-AES) (Meyer and Keliher, 1992; Sah and Miller, 1992).

### Tree Biomass and Annual Nutrient Accumulation

Total biomass increase was calculated as the changes in biomass between January 2012 and January 2013 and between January 2013 and January 2014. Tree biomass changes in season were not determined, however in almond the most vigorous growth occurs between spring and midsummer and then continues with a lower rate (Kester et al., 1996). Based on Kester et al. (1996) and personal communication with Theodore de Jong, Professor Department of Plant Sciences, University of California Davis, we estimated that up to 75% of the total annual biomass increase occurred before harvest and the remaining 25% between harvest and onset of leaf senescence with no biomass accumulation from senescence until January. For calculation purposes we thus allocated 5% of biomass increment by April 10^th^, 60% by June 7^th^ and 75% by August 17^th^ and 100% by November 8^th^ in 2012 and 5% by April 13^th^, 60% by June 26^th^, 75% by August 14^th^, and 100% by November 14^th^ in 2013.

### Seasonal Sampling

Details of sample collection are presented in Table 1. Four trees each from four N treatments were selected and monitored for seasonal nutrient changes in the organs categorized above in 2012. In 2013 N treatment N392 were dropped from the experiment and sample collection was continued for N140, N224, and N309 rates. Wood shavings from trunk, scaffold and canopy branches were collected by boring into the wood as described above. Samples from small branches were collected by cutting several branches, drying, sawing, and grinding as described above. Several large and small roots were dug up using a shovel and axe and categorized into two groups as described above. Roots were placed on ice and brought to the laboratory where they were washed with de-ionized water to remove soil. In 2012 samples were collected on January 26^th^ (dormancy) February 28^th^ (full bloom), March 12^th^ (12 DAFB, fruit set), April 10^th^ (41 DAFB, full leaf expansion), June 7^th^ (98 DAFB, nut fill), August 17^th^ (169 DAFB, harvest), November 8^th^ (249 DAFB, post-harvest), and December 17^th^ (288 DAFB, complete leaf senescence) and in 2013 on March 10^th^ (14 DAFB), April 13^th^ (44 DAFB), June 26^th^ (121 DAFB), August 14^th^ (170 DAFB), October 17^th^ (234 DAFB), November 14^th^ (261 DAFB), and January 14^th^ 2014 (dormant). All samples were ground to pass 0.3 mm screen using a Wiley Mill (Thomas Scientific) and analyzed for nutrients at the ANL.

**TABLE 1:** Sampling dates, crop stage, day after full bloom, and organs sampled for almond trees.

Sampling date	Crop stage	Days after full bloom (DAFB)	Organs analyzed	Purpose
26-January-12	Dormant		Roots, trunk, scaffold, canopy branches	Biomass determination and nutrient analysis
28-Febrauary-12	Bloom		Roots, trunk, scaffold, canopy branches	Nutrient analysis
12-March-12	Fruit set	12	Roots, trunk, scaffold, canopy branches	Nutrient analysis
10-April-12	Full leaf expansion	41	Roots, trunk, scaffold, canopy branches, leaves	Nutrient analysis
7-June-12	Nut fill	98	Roots, trunk, scaffold, canopy branches, leaves	Nutrient analysis
17-August-12	Harvest	169	Roots, trunk, scaffold, canopy branches, leaves	Nutrient analysis
8-November-12	Post-harvest	249	Roots, trunk, scaffold, canopy branches, leaves	Nutrient analysis
17-December-12	Complete leaf senescence	288	Roots, trunk, scaffold, canopy branches, leaves	Nutrient analysis
10-January-13	Dormant			Biomass determination
10-March-13	Fruit set	14	Roots, trunk, scaffold, canopy branches	Nutrient analysis
13-April-13	Full leaf expansion	44	Roots, trunk, scaffold, canopy branches	Nutrient analysis
26-June-13	Nut fill	121	Roots, trunk, scaffold, canopy branches	Nutrient analysis
14-August-13	Harvest	170	Roots, trunk, scaffold, canopy branches	Nutrient analysis
17-October-13	Post-harvest	234	Roots, trunk, scaffold, canopy branches	Nutrient analysis
14-November-13	Leaf fall	261	Roots, trunk, scaffold, canopy branches	Nutrient analysis
14-January-14	Dormant		Roots, trunk, scaffold, canopy branches	Biomass determination and nutrient analysis

### Leaf Samples and Leaf Biomass

Leaf samples from the above-mentioned trees were collected on April 10^th^ (41 DAFB), June 7^th^ (98 DAFB), August 17^th^ (169 DAFB), November 8^th^ (249 DAFB) and December 12^th^ (leaf senescence) of 2012 only. About 1000 leaves from non-fruiting spurs were collected at each sampling time from each tree. The number of leaves in each sample were counted and dried at 60°C for 5 days. After drying each sample was weighed and ground to pass 0.3 mm screen using the Wiley Mill and analyzed for N, P, K, S, B, Ca, Mg, Mn, Zn, Fe, and Cu in the UC Davis ANL. After complete leaf senescence all leaves from the sampled trees were collected and weighed in the field in 2012. A 2 kg sub-sample from each tree was collected and brought to the laboratory where any non-leaf materials were sorted. Samples were then washed to remove any soil material and dried at 70°C for 5 days. After drying samples were weighed and final tree leaf biomass was calculated accounting for the weight loss of the 2 kg sample.

### Size of Nutrient Storage Pool and Nutrient Accumulation in the Season in Tree Organs

The differences in tree nutrient contents during dormancy and the lowest nutrient content in the season (March 12^th^ in 2012 and 2013 for N, and April 10^th^, 2012 and April 13^th^, 2013 for P, April 10^th^, 2012 and March 12^th^, 2013 for K, April 10^th^, 2012 and April 13^th^, 2013 for S and April 10^th^, 2012 and March 12^th^, 2013 for B) were utilized to calculate the size of the nutrient storage pool of the tree. This approach, however, only measures net remobilization as it cannot distinguish between increments in organ N as a result of N input (soil uptake or internal reallocation among organs) and a decrease in organ N as a result of remobilization. Net annual increment in whole tree nutrient content and nutrient distribution in organs was determined by excavation, partitioning and nutrient analysis as described above. The net annual increment in nutrient content represents both new growth and nutrients stored for remobilization in the subsequent year.

### Contribution of Nutrient Remobilization From Leaves to the Storage Pool

The difference in the nutrient content of the entire leaf biomass between harvest and complete leaf senescence was considered as the amount of nutrient remobilized from leaves that was allocated to the storage pool.

Statistical analyses of tree organ biomass, changes in leaf biomass, nutrient storage pool, annual nutrient increment and nutrient sorption from leaves were conducted by analysis of variance using statistical software JMP (John’s Macintosh Project, SAS Inst. Inc., Cary, NC, United States), and means were separated by Tukey HSD at 0.05 level of significance.

## Results

### Perennial and Leaf Biomass

Overall organ growth from first excavation to last excavation (24 months) showed a total biomass increase (all perennial organs) from January 2012 to January 2013 of 3.3, 10.6, 12.4, and 10.8% for N140, N224, N309, and N392 respectively (Table 2). On January 26^th^ 2012 (first excavation), there was a small but non-significant difference in total tree biomass between N rate treatments. On January 10, 2013 total biomass was significantly different between treatments. N309 had the highest biomass (105,447 ± 3139 kg ha^−1^) while N140 had the lowest total biomass (82,094 ± 1317 kg ha^−1^). The total annual perennial biomass increase from January 2013 to January 2014 was 6.8, 6.6, and 6.8% for N140, N224, and N309 respectively, with a total biomass of 112,580 + 5039 kg ha^−1^ at N309 and 87,635 ± 6034 kg ha^−1^ at N140.

**TABLE 2:** Biomass of individual perennial organs and tree total biomass of the excavated trees.

		Biomass of perennial organs (kg ha^−1^)							
	N application			Canopy	Branches > 2.5 cm	Roots < 1 cm	Roots > 1 cm	Total	Annual biomass
Date	(kg ha^−1^)	Trunk	Scaffold	branches	diameter	diameter	diameter	biomass	increase (%)
January 26, 2012	140	7844587*a*	14449417*a*	299823949*a*	14372158*b*	2214126*a*	10611370*a*	794734614*a*	
	224	7880464*a*	124681715*a*	338992144*a*	14107799*b*	2158189*a*	9209525*a*	797215836*a*	
	309	8264622*a*	14214369*a*	408894080*a*	18163113*a*	2304216*a*	9961483*a*	937965208*a*	
	392	8984112*a*	172962443*a*	362741931*a*	18020708*a*	3445746*a*	10137893*a*	941561255*a*	
January 10, 2013	140	7577559*a*	14822465*a*	304131972*b*	15877736*b*	237561*b*	11029114*a*	820941317*c*	3.3
	224	7986408*a*	14127390*a*	359411192*ab*	16799627*ab*	2788284*ab*	105321010*a*	88173295*bc*	10.6
	309	8339172*a*	162131100*a*	45440511*a*	214822909*ab*	254724*ab*	11426210*a*	1054473139*a*	12.4
	392	8563511*a*	17731376*a*	428544517*ab*	201531757*a*	334668*a*	11653944*a*	1043014658*ab*	10.8
January 14, 2014	140	8611639*a*	16371470*a*	310461928*b*	175392190*a*	2711399*a*	113571355*a*	876356034*b*	6.6
	224	8666205*a*	152831752*a*	375163552*ab*	184391649*a*	3094299*a*	11020749*a*	940172349*ab*	6.8
	309	8692276*a*	174762239*a*	477643837*a*	237652926*a*	2936625*a*	11947385*a*	1125805039*a*	6.6

*Mean not connected by the same letter within the same date and organ category are significantly different at 0.05 level of significance using Tukey test.*

Leaf biomass increased with increase in N rate however there was no significant increase in leaf biomass beyond N309 (Table 3). Trees in the N140 treatment produced 1449 kg ha^−1^ leaf biomass while trees in the N224, N309, and N392 treatments had 1695 kg, 1925 kg, and 1990 kg ha^−1^ leaf biomass at complete leaf senescence respectively. The leaves that may have fallen during fruit harvest were not accounted for and hence estimates of total tree leaf biomass provided here may be marginally underestimated (estimated 10–20%).

**TABLE 3:** Seasonal changes in leaf biomass (kg ha^−1^) during the season of 2012.

	Seasonal changes in leaf biomass (kg ha^–^^1^)				
N Application					
(kg ha^–^^1^)	April 10	June 7	August 17	November 8	December 17
140	872 c	1588 c	1648 c	1513 c	1449 c
224	1011 b	1861 b	1935 b	1710 b	1695 b
309	1150 a	2112 a	2189 a	2011 a	1925 a
392	1187 a	2182 a	2258 a	2075 a	1990 a

*Means not connected by the same letter are significantly different at 0.05 level of significance using Tukey test.*

### Time of Nutrient Remobilization From Storage and Reallocation of Nutrients to Storage

**FIGURE 1:**
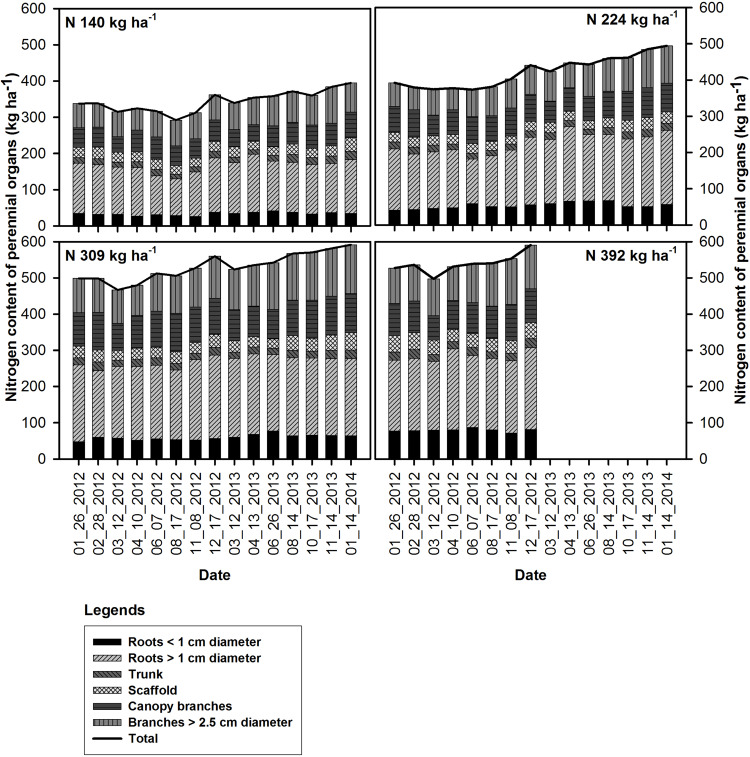
Seasonal changes in the nitrogen content of the perennial organs and mean total tree N content (kg ha^−1^) under N rate treatments in 2012 and 2013.

**FIGURE 2:**
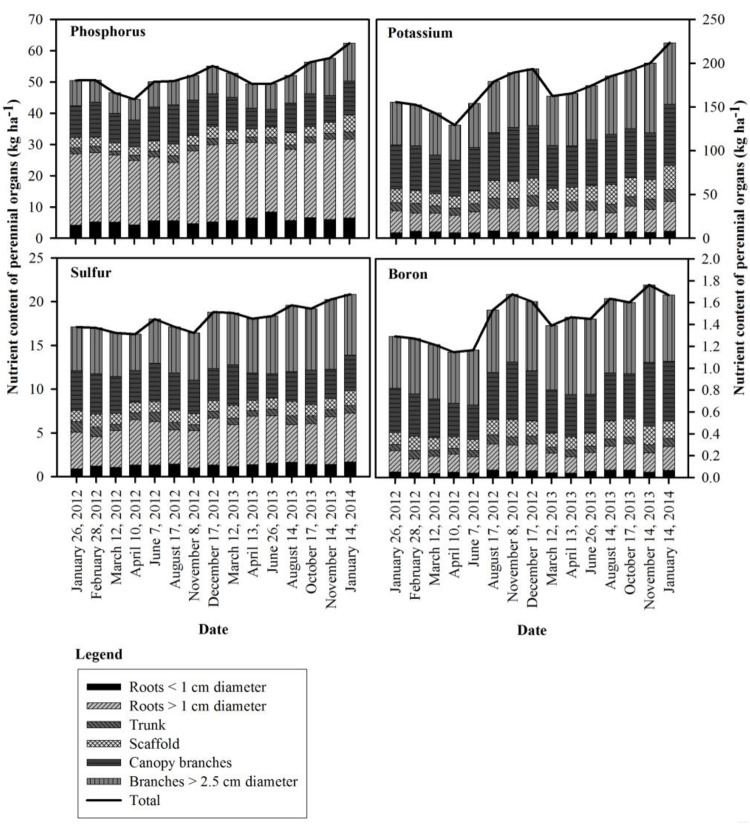
Seasonal changes in phosphorus, potassium, sulfur and boron content of the perennial organs for the 309 kg ha^−1^ N application rate in 2012 and 2013.

The seasonal changes in tree N content for all N rate treatments, and the P, K, S, and B content of the perennial biomass in the N309 rate treatment are shown in Figures 1, 2. The N309 rate was selected to represent P, K, S, and B fluxes as it represented the optimal N fertilization rate in this orchard over the preceding 4 years. A net change in nutrient content of the trees from the beginning to the end of the season (nutrient accumulation) represents nutrients acquired from soil in the current season, however, changes in nutrient content in individual organs was more complex and includes movement of nutrients within organs which were accumulated in the previous season and the acquisition of nutrients from the current year’s uptake. Patterns of change in individual organs and the tree as a whole can be used to determine when remobilization and refilling of nutrient storage pools was occurring.

For all N rate treatments nitrogen content in all perennial organs (whole tree) started declining following on February 28^th^, 2012 (bloom) and was lowest by March 12^th^ in 2012 (12 DAFB) and 2013 (14 DAFB). It then increased after April 10^th^, 2012 (41 DAFB) and April 13^th^, 2013 (44 DAFB, leaf expansion) and continued through the season until December 17^th^ 2012 and January 14^th^ 2014 except for the very deficient N rate N140 in 2012 which remained low during season until harvest (169 DAFB) then increased after harvest until leaf senescence (249 DAFB).

Seasonal patterns of whole tree nutrient content differed from N for P, K, S and B, with the lowest whole tree nutrient contents observed on April 10^th^, 2012 (41 DAFB) for P, K, and B. In 2013, P and S were lowest by April 13^th^ (44 DAFB), K and B by March 12^th^ (14 DAFB). Tree P content started declining on February 28^th^ (bloom) and continued until April 10^th^ in 2012 (41 DAFB) and April 13^th^ in 2013 (44 DAFB, full leaf expansion) and then increased gradually and reached its peak on December 17^th^, 2012 and January 14^th^, 2014 (dormancy). The greatest increase in tree P content occurred between June 7^th^ (98 DAFB) and December 17^th^ (dormancy) in 2012 and June 26^th^, 2013 (121 DAFB) and January 14^th^, 2014 (dormancy). K remobilization commenced on February 28^th^ (bloom) and continued until April 10^th^ (41 DAFB) and March 12^th^ (14 DAFB) in 2012 and 2013 respectively. Tree K content then increased strongly between the end of April 10^th^ (41 DAFB) and August 17^th^ (169 DAFB) in 2012, with little subsequent increase in tree K content by December 17^th^, 2012 (dormancy). In 2013 there was consistent increase in K content from April 13^th^ (44 DAFB) which continued until January 14^th^, 2014 (dormancy). Remobilization of S started following February 28^th^ (bloom) and tree S content reached its lowest point on April 10^th^ (41 DAFB) in 2012. S accumulation in the perennial organs changed during 2012 season with two periods of remobilization of S from the perennial organs – one by April 10^th^ (41 DAFB, full leaf expansion) and another by August 17^th^, 2012 (169 DAFB, harvest). In 2013 S content of perennial organs declined until April 13^th^, 2013 (44 DAFB, full leaf expansion) and increased thereafter until January 14^th^, 2014 (dormancy). Tree boron content started declining following February 28^th^ (bloom) and continued until April 10^th^ (41 DAFB) and March 12^th^ (14 DAFB) in 2012 and 2013. In 2012, there was little change in tree B content between April 10^th^ (41 DAFB) and June 7^th^ (98 DAFB) and then there was a rapid increase in tree B content after June 7^th^ that reached a peak by November 8^th^ (249 DAFB). A similar pattern was observed in 2013, where B content greatly increased between June 26^th^ (121 DAFB) and August 14^th^ (170 DAFB, harvest).

**FIGURE 3:**
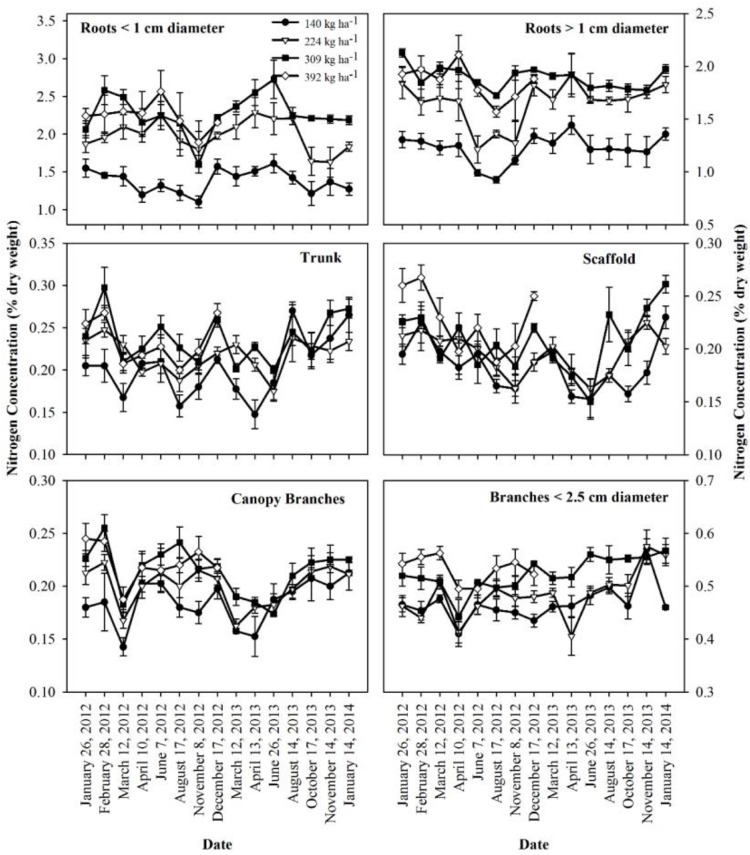
Nitrogen concentrations in perennial tissues as affected by N application in 2012 and 2013 seasons. Data shows mean and standard error of the mean.

The concentration of N in perennial organs changed through the season (Figure 3). The magnitude of changes varied across N treatments with largest fluctuations evident for N309 and N392 in trunk, scaffold and branches < 2.5 cm diameter. The organs that held the largest amount of remobilizable nutrient reserves (roots > 1 cm diameter and canopy branches) exhibited the largest and most synchronized changes. Besides seasonal variation, year to year variation was also evident where fluctuations in N concentration in roots > 1 cm diameter and canopy branches in 2013 were smaller than 2012. Nitrogen concentrations in roots > 1 cm diameter, scaffold branches and canopy branches exhibited a strong decrease in total content (Figure 1) and concentration (Figure 3) during the period of dormancy to that of leaf out, suggesting that these organs were the major N-source for early N demand. Subsequently, trunk and scaffold branches, roots < 1 cm diameter and roots > 1 cm diameter exhibited a strong decline in N concentrations on June 7^th^ and June 26^th^ (nut fill) in 2012 and 2013 respectively and on August 17^th^ and August 14^th^ (harvest) in 2012 and 2013 respectively, which represents a high fruit N demand. Subsequent to fruit harvest (August 17^th^, 2012 and August 14^th^, 2013), N concentrations in all organs increased.

### Size of Nutrient Storage Pool, Perennial Organs Used for Storage and Annual Nutrient Accumulation in Perennial Tissues

Whole tree remobilizable N, P, K, S, and B storage pools in the perennial tree biomass for all N rates was calculated as the difference between the nutrient content at the beginning of the year and the lowest total nutrient content during the growing season (Table 4). Remobilizable N storage was significantly different between N rate treatments in both 2012 and 2013. N storage varied between N treatments with lowest N stored (28.5 ± 2.1 kg ha^−1^ in 2012 and 26.5 ± 4.8 kg ha^−1^ in 2013) in the N140 and the highest (44.4 ± 4.0 and 37.5 ± 5.7 kg ha^−1^ in 2012 and 2013 respectively) for N309. Remobilized N from storage contributed to 24 and 33% of the annual organ N demand and was equivalent to 100% of N demand between February 28^th^ (bloom) and March 12^th^ (fruit set) in 2012 and 2013.

**TABLE 4:** Total nitrogen, phosphorus, potassium, sulfur, and boron storage pool (kg ha^−1^) in perennial biomass of almond trees under differential N application rates calculated as nutrient content when tree is dormant minus lowest nutrient content in perennial tissues during the season.

		Storage Pool (kg ha^–^^1^)				
	N Application					
Year	(kg ha^–^^1^)	N	P	K	S	B
2012	140	28.5*b*	9.45*a*	22.9*a*	1.57*ab*	0.09*b*
	224	32.9*b*	4.95*a*	24.8*a*	1.36*b*	0.15*a*
	309	44.4*a*	7.53*a*	28.4*a*	2.56*a*	0.18*a*
	392	40.3*a*	6.67*a*	21.4*a*	0.89*b*	0.14*ab*
	Average		7.2	24.4	1.6	0.10
2013	140	26.5*b*	4.63*a*	30.0*a*	0.77*b*	0.22*a*
	224	34.6*a*	7.40*a*	35.4*a*	1.71*a*	0.28*a*
	309	37.5*a*	8.50*a*	27.0*a*	1.38*a*	0.22*a*
	Average		6.8	30.8	1.3	0.2

*Means not connected by the same letter within the same year are significantly different at 0.05 level of significance using Tukey test.*

There were no significant differences in P and K storage with N rate treatments. S storage was significant different between N treatments in 2012 and 2013, however there was no consistent relationship between S storage and N application. B storage was affected by N treatments in 2012 only. P, K, S, and B storage averaged 7.2 and 6.8 kg ha^−1^, 24.4 and 30.8 kg ha^−1^, 1.6 and 1.3 kg ha^−1^ and 0.1 and 0.2 kg ha^−1^ in 2012 and 2013 respectively across all treatments. The storage of other nutrients (Mn, Zn, Fe, and Cu) could not be detected as no net changes in nutrient pool sizes were observed.

### Perennial Tissues Used for Nutrient Storage

The amount of nitrogen that was remobilized from each perennial organ for N rate treatments is shown in Table 5. All organs have the capacity to store and remobilize N, however, based upon the size of the pool, roots > 1 cm diameter and canopy branches were the major storage organs for all N rates and in both 2012 and 2013. Small amounts of N were also stored in the trunk and scaffolds as well as in branches < 2.5 cm diameter. Nitrogen application positively influenced the amount of N storage in roots > 1 cm diameter and canopy branches.

**TABLE 5:** Nitrogen stored (kg ha^−1^) in perennial tree organs (nutrient content when tree is dormant minus lowest nutrient content in perennial tissue during season) for almond trees with differential N application rates.

		Nitrogen Rate (kg ha^–^^1^)			
Year	Organ type	140	224	309	392
2012	Roots < 1 cm diameter	4.73.7	1.31.3	0.00	3.11.8
	Roots > 1 cm diameter	8.52.8	12.92.6	14.22.4	5.10.7
	Trunk	2.91.1	2.21.3	2.50.7	4.32.2
	Scaffold	0.40.4	1.31.3	5.00.9	6.54.2
	Canopy branches	11.31.9	15.33.5	17.91.5	20.95.0
	Branches < 2.5 cm diameter	0.70.7	0.00.0	4.82.9	0.50.5
2013	Roots < 1 cm diameter	3.21.8	2.01.2	0.30.3	
	Roots > 1 cm diameter	10.22.7	14.31.5	12.00.7	
	Trunk	2.81.0	0.40.2	4.80.4	
	Scaffold	0.40.4	0.00.0	4.70.7	
	Canopy branches	12.23.3	16.22.3	13.11.5	
	Branches < 2.5 cm diameter	0.60.6	1.71.2	5.92.2	

*Data shows the mean and standard error of the mean.*

P, K, S, and B storage in perennial organs is shown in Table 6. P was mostly stored in roots > 1 cm diameter, canopy branches and branches < 2.5 cm diameter. Trunk and scaffolds also stored small amount of P that was remobilized to support the annual tissues early in the season. Potassium was mostly stored in canopy branches, branches < 2.5 cm diameter and roots > 1 cm diameter. K was also stored in trunk and scaffolds in smaller amounts. For the standard N rate (N309) the storage pattern was: canopy branches > branches less than 2.5 cm diameter > roots greater than 1 cm diameter > scaffold in 2012 and 2013. Most of the S was stored in canopy branches and branches < 2.5 cm diameter followed by roots > 1 cm diameter. Boron was stored in canopy branches and branches < 2.5 cm diameter and roots > 1 cm diameter.

**TABLE 6:** Phosphorus, potassium, sulfur, and boron storage (kg ha^−1^) in tree perennial organs (nutrient content when tree is dormant minus lowest nutrient content in perennial tissue during season) for almond trees for 309 kg ha^−1^ N application rate.

Nutrients stored (kg ha^–^^1^)					
Year	Organs type	P	K	S	B
2012	Roots < 1 cm diameter	0.40.2	0.30.2	00	0.080.01
	Roots > 1 cm diameter	3.21.2	6.73.8	0.30.2	0.040.02
	Trunk	0.20.1	1.30.7	0.40.1	0.010.003
	Scaffold	0.50.1	2.10.5	0.10.0	0.0030.003
	Canopy branches	1.70.1	9.31.9	0.90.1	0.100.003
	Branches < 2.5 cm diameter	1.50.7	8.85.6	0.90.4	0.030.02
2013	Roots < 1 cm diameter	00	0.30.2	0.10.1	0.020.01
	Roots > 1 cm diameter	1.91.0	4.81.5	0.30.2	0.070.02
	Trunk	0.30.0	1.70.7	0.10.0	0.020.01
	Scaffold	1.30.5	3.30.7	0.20.1	0.020.01
	Canopy branches	3.90.8	11.25.4	0.40.2	0.060.04
	Branches < 2.5 cm diameter	1.20.4	5.83.7	0.30.2	0.060.04

*Data shows the mean and standard error of the mean.*

### Annual Nutrient Accumulation in Perennial Biomass

The total annual nutrient accumulation in the perennial biomass of trees was calculated from the difference in whole tree nutrient content between January 2012, December 2012 and January 2014 (Table 7). Nitrogen accumulation in perennial organs was significantly affected by N application rate and was maximum (61.2 ± 7.8 kg ha^−1^) for N392 and minimum (25.8 ± 4.7 kg ha^−1^) for N140 treatment in 2012. In 2013 maximum N accumulation (57.2 ± 5.2 kg ha^−1^) was observed for N rate N309 and minimum (40.4 ± 4.7 kg ha^−1^) for N rate N140. There was no consistent trend in the accumulation of P, K, and S with N application. For the N309 which resulted in optimum yield, the accumulation of P, K, and S was 4.8 ± 2.1 kg, 37.9 ± 2.0 kg, and 1.7 ± 0.6 kg ha^−1^ in 2012 and, 7.3 ± 5.4 kg, 29.8 ± 2.2 kg, and 2.0 ± 0.6 kg ha^−1^ respectively in 2013.

**TABLE 7:** Annual nutrient increment in perennial biomass calculated as the difference in nutrient content of the tree at the beginning and the end of seasons.

		Annual total nutrient accumulation in perennial tissues (kg ha^–^^1^)			
	N Application				
Year	(kg ha^–^^1^)	N	P	K	S
2012	140	25.84.7*b*	2.71.0*b*	18.37.6*c*	0.80.4*c*
	224	59.54.5*a*	7.92.9*a*	22.53.0*c*	2.00.6*b*
	309	56.06.3*a*	4.82.1*ab*	37.92.0*b*	1.70.6*bc*
	392	61.27.8*a*	4.61.1*ab*	52.510.4*a*	3.60.6*a*
2013	140	40.44.7*b*	9.41.8*a*	20.82.1*b*	1.60.6*a*
	224	46.37.0*ab*	6.63.0*b*	19.27.1*b*	2.10.8*a*
	309	57.25.2*a*	7.35.4*ab*	29.82.2*a*	2.00.6*a*

*Means not connected by the same letter within the same year are significantly different at 0.05 level of significance using Tukey test.*

### Nutrient Remobilization From Leaves to Storage Pool

Nitrogen, P, K, S, and B remobilization from leaves to the storage pools, the seasonal maximum and minimum concentrations and leaf biomass were monitored in 2012 only and are shown is Tables 3, 8, 9 respectively. N remobilization from leaves increased as N application increased. Maximum N remobilization (15.5 ± 0.6 kg ha^−1^) was observed for N309 and minimum N remobilization (11 ± 0.9 kg ha^−1^) for N140. Phosphorous remobilization was similar for all N rates at an average of 1.0 ± 0.2 kg ha^−1^. The highest K remobilization from leaves (20 ± 5.6 and 20 ± 2.6 kg ha^−1^) was recorded for the N140 and N224 treatments while the lowest K remobilization (8.9 ± 1.1 kg ha^−1^) was observed for the N309 rate. Sulfur remobilization from leaves increased significantly as N application rate increased and ranged from 0.2 ± 0.1 to 0.8 ± 0.2 kg ha^−1^ for N140 versus N392 rate. Boron remobilization averaged at 0.04 ± 0.01 kg ha^−1^ across N rate treatments. The concentration of N, P, K, S, and B declined during the season with highest concentrations observed in April and lowest concentrations during leaf senescence (Table 9). Leaf biomass increased as N rate increased and was maximum for N392. Leaf biomass increased during the season for all treatments with lowest biomass on April 10^th^ and maximum on August 17^th^ which then deceased until December 17^th^ (leaf senescence). On August 17^th^, N392 produced maximum (2258 ± 41 kg ha^−1^) and N140 produced minimum (1648 ± 69 kg ha^−1^) leaf biomass.

**TABLE 8:** Resorption of N, P, K, S, and B (kg ha^−1^) from leaves to the storage pool under different nitrogen application rates, calculated as the difference between leaf nutrient content at harvest and at leaf senescence.

	Nutrient resorption (kg ha^–^^1^)				
Nitrogen application					
(kg ha^–^^1^)	Nitrogen	Phosphorus	Potassium	Sulfur	Boron
140	110.9*b*	1.00.2*a*	205.6*a*	0.20.1*b*	0.030.01*a*
224	11.81.5*b*	0.980.1*a*	202.6*a*	0.20.1*b*	0.040.01*a*
309	15.50.6*a*	0.970.1*a*	8.91.1*b*	0.40.1*b*	0.040.01*a*
392	14.82.3*a*	1.00.2*a*	10.82.5*b*	0.80.2*a*	0.030.01*a*
Average		1.00.2	14.93.0	0.40.1	0.040.01

*Data represents mean and standard error. Means not connected by the same letter are significantly different at 0.05 level of significance using Tukey test.*

**TABLE 9:** Mean seasonal maximum and minimum concentrations of N, P, K, S, and B in leaves in 2012.

	Nitrogen (%)		Phosphorus (%)		Potassium (%)		Sulfur (ppm)		Boron (ppm)	
	
N Supply										
(kg ha^–1^)	April 10	December 17	April 10	December 17	April 10	December 17	April 10	December 17	April 10	December 17
140	2.89*c*	1.45*c*	0.25*a*	0.09*a*	2.73*a*	1.41*b*	2379*a*	1667*a*	35*a*	29*a*
224	3.19*bc*	1.78*b*	0.26*a*	0.10*a*	2.60*a*	1.59*ab*	2603*a*	1800*a*	37*a*	29*a*
309	3.49*ab*	1.80*b*	0.27*a*	0.10*a*	2.28*a*	1.61*ab*	2285*a*	1667*a*	38*a*	29*a*
392	3.52*a*	2.03*a*	0.25	0.10*a*	2.15*a*	1.73*a*	2530*a*	1700*a*	34*a*	28*a*

*Means not connected by the same letter are significantly different at 0.05 level of significance using Tukey test.*

## Discussion

In almond, flowering generally precedes leaf out and thus soil nutrient uptake may be limited at this time due to lack of transpiration, abundance of remobilized N and restricted root activity. Almond annual tissues (leaves and fruits) have higher N demand early in the season (Muhammad et al., 2015) whereas root growth begins after fruit set and leaf out (Olivos and Brown, unpublished data). Flowering, fruit set and initial leaf growth must therefore depend predomiantly on stored nutrients in the tree perennial structure.

Nitrogen remobilization, as determined by a net decrease in N in perennial organs, started at bloom (February 28^th^ and continued until fruit set March 12^th^) for all N rates, then there was an increase in the N content of the perennial tissues except for the low N rate treatment (N140) which showed a lower N content in perennial tissues at kernel fill in 2012 (August 17^th^) which represents a high N demand time. This suggests that N uptake from the N140 treatment was not sufficient to meet the N demand of the crop and tree growth. Net uptake of N from soil only commenced at 70% leaf out which coincided with the beginning of root growth observed under mini-rhizotrons in the same field (Olivos and Brown, unpublished data). In other perennial crops, N storage in the perennial organs has been reported to support the growth before active uptake from soil begins. In pecan, Acuna-Maldonado et al. (2003) observed a decline in tree N content until crop demand was met by uptake which occurred at the completion of leaf expansion. Rosecrance et al. (1996) reported soil N uptake in late spring in alternate bearing high yield years in pistachio while the early season tree N demand was apparently met by storage. In this investigation, N content of perennial biomass started increasing early, after April 10^th^ and April 13^th^ in 2012 and 2013 and continued until leaf fall. In these experiments, we only analyzed total N content of the tree and hence it was not possible to distinguish changes in remobilizable N, new N uptake and N utilized for the growth of perennial organs. The data suggests that filling of the remobilizable reserves started before harvest for N309 and N392 as evidenced by the increase in the nutrient content of canopy branches and branches < 2.5 cm diameter while the filling of reserves in the roots occurred later in the season (Figure 1). The greatest increase in tree N content at N140 and N224 applications occurred between harvest and leaf fall in 2012 and was predominantly a consequence of an increase in tissue N concentrations (Figure 3). In 2013 there was a gradual increase in N content of the perennial organs after April 13^th^ under all N rates and continued during the season until leaf fall.

The increase in N content between early November and mid-December (end of leaf senescence) was a result of N remobilization from senescing leaves and root N uptake from soil. Senescing leaves contribute to the nitrogen storage pool in amounts varying from 9 to 87% in different species (Millard and Grelet, 2010). Presuming the same amount of N storage occurred at the end of the 2013 season as at the end of 2012, N remobilization from leaves in 2012 may have contributed to 39, 36, 35, and 47% of the estimated storage pool for the N140, N224, N309, and N392 application rates respectively whereas in 2013 the contribution of N from remobilization was 42, 34, and 41% for N140, N224, and N309 respectively. While this represents a significant contribution to the N demand in the early stages of almond growth until the end of leaf expansion, the overall contribution of leaf remobilization to the annual N budgets would represent less than 5% of the total annual nutrient demand in an average yielding year in which 4,500 kg ha^−1^ nuts would be expected (Muhammad et al., 2015, 2018).

In contrast to N, remobilization of stored P, K, and S continued until completion of leaf expansion and thereafter their content increased in perennial tissues as their uptake from soil increased. Lower temperatures in early spring may have reduced P, K, and S uptake and for this reason reserves may have been depleted over a longer time (Figure 2). Phosphorous uptake and availability increases with increasing air and soil temperature (Sheppard and Racz, 1984; Ercoli et al., 1996). Similarly low spring temperatures have been shown to reduced K uptake in corn (Ching and Barber, 1979). The increase in P content was steady with a small increase in accumulation near the beginning of leaf senescence. Significant amounts of P and S were contributed by remobilization from senescing leaves (Table 7) with the majority of P and S coming from in-season uptake from soil. In June 2012 and 2013, foliar boron was applied which could have increased tree boron content. The storage and remobilization of Ca, Mg, Zn, Mn, Fe, and Cu could not be clearly determined for two apparent reasons: (1) these nutrients were not stored and remobilized, or (2) the methods used were not adequately sensitive to detect the low quantities involved.

The nutrient storage pool was determined as the difference between nutrient content of the perennial biomass at dormancy and the lowest nutrient content during the season. A similar approach was used by Rosecrance et al. (1998) to determine nutrient storage in mature pistachio trees. We observed an increase in the N storage pool in the perennial tree biomass with increased N application rates. The increase in the N storage pool with increasing N application rate was mainly due to an increase in tree biomass (Table 4) and N concentration in different perennial organs of the tree (Figure 3). Similar results were reported by Miller (1995) where increase in biomass increased storage capacity and internal nutrient cycling of trees. After 4 years of differential N treatments, tree biomass increased as N application increased (Table 2).

For the N309 rate, roots > 1 cm diameter and canopy branches remobilized 14.2 ± 2.26 kg ha^−1^ and 17.93 ± 1.53 kg ha^−1^ N respectively between bloom and 70% leaf out and that represented 8 and 10 and 11% of the total increment in annual tissue N until full leaf expansion in 2012 and 2013 respectively. Remobilized N represented 24 and 33% of the total N accumulation in 2012 and 2013 in annual tissues between bloom and spur leaf expansion. Varying amounts of N storage have been reported in tree species ranging from 50% of the total tree perennial N content for walnut (*Juglans regia* L.) (Weinbaum and Van Kessel, 1998) to 6–38% for alternate bearing high yield year and low yield years for pistachio (Rosecrance et al., 1998), to 11–16% of the tree N in pecan (Acuna-Maldonado et al., 2003). Weinbaum et al. (1987) using ^15^N estimated that 50% of the tree N demand was contributed by N from storage pools. This number is much higher than we have observed from the nutrient budgets assembled in our current study, where stored N accounted for 10% of the N demand of annual tissues (Muhammad et al., 2015, 2018). In their study Weinbaum and co-workers only analyzed leaves and fruits to calculate the contribution of storage pools and any unlabeled N present in fruit and leaves was considered a contribution from storage pools. They did not account for the contribution of unlabeled N present in soil or that could be contributed from mineralization of soil organic matter. The combination of tree excavation and coring to determine tree biomass and seasonal changes in nutrient content used here and the determination of whole tree N budgets provided more accurate information on the seasonal fluxes of nutrients in perennial as well as annual tissues.

Roots and trunks have been reported as the major N storage organs in trees (Wetzel et al., 1989; Coleman et al., 1992; Millard and Grelet, 2010; Carranca et al., 2018). The amount of nutrient stored in an organ depends on the nutrient concentration and the total standing biomass of the organ. In almond, roots > 1 cm diameter were the major site of N storage followed by canopy branches. The storage of nutrients in the trunk and scaffold was quite small due to the lower concentration present (Figure 3). Branches < 2.5 cm diameter had highest biomass but had lower concentration and stored N.

Most of the P reserves came from uptake while remobilization from senescing leaves also contributed marginally to storage. Research shows both uptake from soil and remobilization from leaves contribute to P storage (Rosecrance et al., 1998). Most of the P and K was stored in canopy branches followed by branches < 2.5 cm diameter and roots > 1 cm diameter. A similar pattern of P and K storage has been reported by Rosecrance et al. (1998) in pistachio. About 50% of the stored S was in branches < 2.5 cm diameter. In beech (*Fagus sylvatica*) 47% of the S was stored in branches and 16% in the trunk (Herschbach and Rennenberg, 1996). Boron demand of annual tissues early in the season was met by B remobilization. Mobility of B is species dependent, and in *Prunus* it is phloem mobile as a result of boron sorbitol complexation (Brown and Hu, 1996).

No substantial nutrient storage was found in roots < 1 cm diameter and a similar effect was observed in pecan (Acuna-Maldonado et al., 2003). Tree N content was lowest by March 12^th^ when most of the stored N was utilized by the tree. By this time uptake of nutrients from soil had started as the tree was 70% leaf out and the uptake of nutrients from soil probably increased the concentrations of nutrients in the fine roots since concentrations were greater by March 12^th^ than the trees were dormant thus resulted in increased nutrient content in fine roots (Figure 3).

## Conclusion

N, P, K, S, and B stored in perennial tissues is the primary source of nutrients for flowering, fruit set and early leaf and fruit growth from flowering until 70% leaf out has been achieved. The importance of stored nutrients for this phase of tree growth has important implication for prior season fertilization strategies. In areas of high early spring rainfall, the knowledge of nutrient storage can be used to adjust N fertilization timing and avoid N leaching from fertilizers. Almond trees were observed to store physiologically significant amounts of N, P, K, S, and B in canopy branches, roots > 1 cm diameters and branches < 2.5 cm diameter. Soil uptake from late leaf out until late fruit maturity was the major source of these nutrients allocated to these storage pools, while remobilization from senescing leaves also contributed significantly.

## Data Availability Statement

The datasets generated for this study are available on request to the corresponding author.

## Author Contributions

SM: conducting research, data collection and analysis, and writing manuscript. BS: treatment application and data collection. BL: tree excavation and data collection. DS: proposal writing, data collection, and review. SS: applied treatments and collected samples and data. KS: proposal writing and data collection. PB: research management and writing.

## Conflict of Interest

The authors declare that the research was conducted in the absence of any commercial or financial relationships that could be construed as a potential conflict of interest.
